# Challenges of carbon emission reduction by the workshop education pattern

**DOI:** 10.1016/j.heliyon.2023.e13404

**Published:** 2023-02-10

**Authors:** Na An, Chenyu Huang, Yanting Shen, Jinyu Wang, Jiawei Yao, Philip F. Yuan

**Affiliations:** a College of Architecture and Urban Planning, Tongji University, Shanghai, 200092, China; b School of Architecture and Art, North China University of Technology, Beijing, 100144, China; c Key Laboratory of Ecology and Energy-saving Study of Dense Habitat (Tongji University), Ministry of Education, Shanghai, 200092, China

**Keywords:** Workshop, Carbon footprint, Transport mode, Online/in-person, Learning efficiency

## Abstract

The COVID-19 pandemic has forced many conferences and educational events to shift from in-person to online, significantly reducing the carbon footprint associated with these activities. Workshops are a common pattern of thematic learning at the university level, usually involving a series of activities, such as gathering, learning, and dining, for participants from different regions. However, unlike a three-day conference, workshops usually last for seven days or more, resulting in a non-negligible carbon footprint. To resolve this challenge, we have developed a model that provides recommendations for minimizing the carbon footprint of workshops. Using data from the DigitalFUTURES International Workshop on architecture education at Tongji University in China, we calculated the carbon footprint of scenarios with varying workshop durations, participation modes, and transportation methods. Our results show that online workshops can reduce the carbon footprint by up to 88% compared to in-person workshops. Hybrid workshops, which combine online and in-person participation, can also lead to significant carbon reductions, with a 46% online participation rate resulting in an 82% reduction in carbon footprint. However, we recommend that in-person participation be maintained to ensure efficient learning and effective communication. Our work provides a sustainable solution for organizing future workshops with a reduced carbon footprint.

## 1 Introduction

The Earth is experiencing a round of climate change characterised by global warming. Current greenhouse gases in the Earth's atmosphere have gone from 280 ppm in pre-industrial times to 410 ppm in 2019, far exceeding the safe threshold for atmospheric carbon dioxide concentrations (350 ppm) [1]. Dramatic climate change prompts countries to take urgent action [2]. The carbon footprint describes the total greenhouse gas emissions caused by an activity, product or population [3]. It is also a measure of the impact of climate change [4], greenhouse gas and carbon management, and is usually measured in kilograms of CO_2_ equivalent produced [5]. The United Nations 2019 Emissions Gap Report states that global annual carbon emissions need to be reduced by 7.6% to limit the average temperature rise to 1.5 °C as stipulated in the Paris Agreement [6]. However, at the current rate of emissions, there is a high probability that temperatures will break 1.5 °C in the next five years [7].

Conferences serve as an essential part of a researcher's work. Academic conferences can facilitate the dissemination of ideas, build collaborative relationships for scholars, and provide educational, training, and career opportunities [8]. The study found that even small conferences have some carbon emissions [9]. The carbon emissions of a short 3-day conference are approximately equal to the weekly carbon emissions of six average US households [9,10], while a week-long conference would generate the weekly carbon emissions of the entire city of Edinburgh, UK [11]. Round-trip transportation contributes the majority of the carbon footprint of the conference [12]. In contrast to other types of travel, attendees are free to choose how they want to travel to the conference [13], which opens up the possibility of reducing the conference's carbon footprint [14,15].

The COVID-19 pandemic presents significant challenges and opportunities for economic, social and educational development [16]. In response to the COVID-19 pandemic, countries have adopted various control strategies to reduce the spread of the epidemic [17], such as international isolation and reduced mobility [18]. These factors may reduce participants' travel to countries with less research activity, such as those not in the top 10 countries the Nature Index defines [19]. The COVID-19 pandemic has prompted many conferences to move online [16,20], and recent studies comparing online and in-person research. Recent studies have compared the carbon footprint of in-person and online conferences [9,21,22], calculating the carbon reduction potential of online and in-person meetings. Astudillo et al. and Desiere et al. suggest that online meetings' per capita carbon emissions range from 0 to 5.87 kg CO2, and online conferences' carbon footprint is at least 97 to 200 times smaller than in-person conferences [10,23].

Scholars have been proposing many policies to reduce the carbon footprint of conferences, such as meeting location optimisation [10,23], promoting land transport [24], increasing the carbon tax on flights [21] and hybrid meeting models [25]. However, some scholars consider online meetings to be an utterly carbon-reducing meeting model, ignoring the energy consumption of a single person during a videoconference, the network infrastructure consumption of videoconferencing, and other greenhouse gas emissions [9,26]. In terms of indoor energy consumption, the total single-person energy consumption in an online meeting is likely to be higher than an in-person meeting's energy consumption.

Many scholars have analyzed the carbon emissions of online conferences, but less attention has been paid to workshops, and there is no detailed research on how longer-term workshops reduce the environmental impact of carbon footprints. Workshops, a common pattern of learning at the university level, allow learners to gain exposure to other knowledge. Workshops enable learners to travel to different countries to learn knowledge and summarise it in educational activities [27]. Workshops are more interactive than regular academic conferences, but they may bring problems such as long duration and high energy consumption. Accordingly, the present study utilizes the "AI for Carbon Neutral Cities" subproject as an illustrative example from the 2022 DigitalFUTURES International Workshop Series (https://digitalfutures.world/) held at Tongji University, China. Our analysis involves a comprehensive life-cycle assessment of both in-person and online workshop formats, with the aim of providing a more sustainable solution for organizing future workshops.

Standard methods for calculating carbon footprints include the input-output method [28], the IPCC method [29], the LCA method [30] and the carbon footprint calculator method [31]. This paper uses the LCA method to calculate the carbon footprint generated during the workshop. The LCA method, also known as Life Cycle Assessment, is a method to assess the environmental impact and potential impact of all the inputs and outputs of a product, service, process or activity throughout its life cycle. The LCA method is a more detailed and accurate process and is suitable for calculating carbon footprints at a micro level.

The LCA method has been used to calculate the carbon footprint of the meeting life cycle. However, studies mainly focused on quantifying the carbon footprint of in-person meetings [24,32] and have paid little attention to other impact categories, focusing mainly on the carbon footprint calculation of round-trip transportation [14,33], also considering the life cycle stages of meeting catering, accommodation and transportation [9,23]. The conferences carbon footprint ranges from 92 to 3540 kg per capita, depending on the duration, size and location of the meeting [26], and almost all of these studies consider transportation as the significant share of carbon emissions from meetings (ranging from 50% to 90%) [22], especially from air travel [34]. It is also related to the location of the meeting, where dense train links to other cities may encourage attendees to choose more carbon-reducing land transport. Scholars' opinions differ regarding conference catering and accommodation. Bossdorf et al. suggest 18% and 13% of the total carbon footprint for accommodation and food, respectively [20], While Astudillo and Azarijafari suggest that food and accommodation account for only 1% and 2% of the total carbon footprint [22].

The DigitalFUTURES International Workshop is a globally recognized community of architects and urban planners, established by the College of Architecture and Urban Planning at Tongji University. In collaboration with organizations such as DigitalFUTURES community and Union International des Architectes, the workshop offers an integrated online and in-person educational platform. The 2022 workshop encompasses a diverse range of events, including lectures, conferences, workshops, and exhibitions, with the topic of "One Planet". One of the key subprojects, "AI for Carbon Neutral Cities," is a design and research initiative that explores how artificial intelligence can facilitate the realization of carbon-neutral cities, particularly in light of the challenges posed by climate change and the increasing carbon emissions in urban areas. Owing to the COVID-19 pandemic, the workshop was conducted online.

**Fig. 1:**
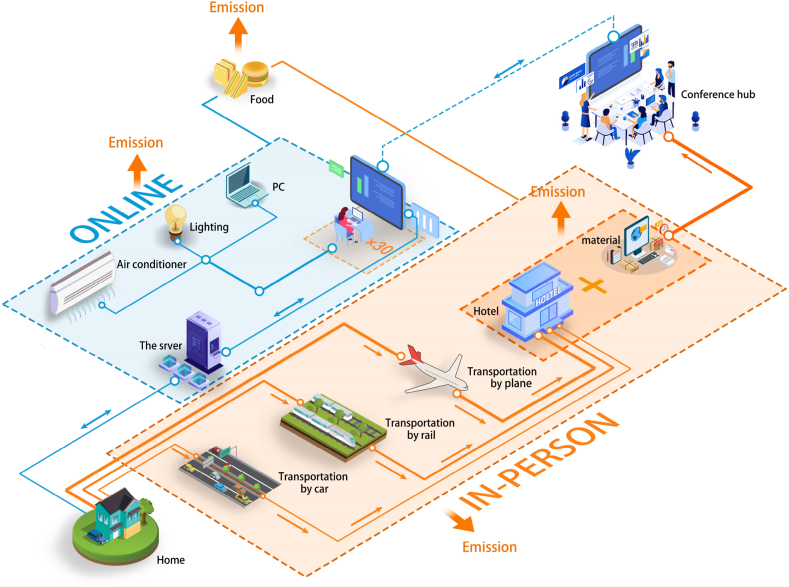
Schematic diagram of the participant path for in-person and online participants.

The "AI for Carbon Neutral Cities" subproject had 60 participants, with 30 individuals attending a 7-day session focused on carbon emission reduction and AI theory teaching, as well as technical guidance. Another 30 participants attended only the first day of teaching and the 3-day Young Scholars Forum. The workshop's carbon footprint is shown in Fig. 1, with the purple line indicating the online pathway and the green line indicating the in-person pathway of the workshop. In addition, we considered eight scenarios with different modes of transport over different distances (900 km, 1000 km, 1200 km, 1400 km, 1600 km, and 2000 km using train transport, all train transport and all airplane) to examine the balance between online, in-person and hybrid carbon footprints. The study provides the public with the advantages and disadvantages of different workshop days and transport modes, clarifying the importance of improving energy efficiency and resource use of information and communication technologies (ICT). Finally, the analysis can assess the environmental impact of attendees with different timings and modes of transport and recommend that event organisers set reasonable workshop participation lengths and traffic levels, providing a sustainable carbon reduction policy for future workshops.

## 2 Materials and methods

### 2.1 Data sources

This paper collects information on participation in the workshop from 26 June to July 2, 2022. The data collected includes basic information on the participants, transport and environmental information, such as the number and type of participants (e.g., students and industry personnel), geographical location, and source organisations. The data sources were participant registration information on the workshop website and questionnaires. The questionnaire consisted of an essential environmental survey of the participants, a daily energy consumption survey, and a site improvement suggestion to collect qualitative data. The questionnaires are shown below (Table 1). 30 participants, 30 regular participants and 5 teaching assistants participated in this workshop, among which 3 questionnaires were invalid, and 62 data were collected, with an efficiency rate of 95%. The survey is available in the supplementary information.

**Table 1:** Content of data collection.

Questionnaire name	Content covered
Registration Information	Name, gender, age, education, occupation
Base carbon footprint	Name, the reason for choosing in-person, mode and reason for participating in intercity and intra-city transport, mode of accommodation; reason for choosing online, workplace, equipment, lighting, cooling mode and wattage, energy efficiency
Daily Carbon Footprint	Working hours, Lighting hours, air conditioning use hours
Camp Feedback	Learning efficiency, communication effectiveness, Teaching efficiency, competence performance, financial costs, perception of teaching effectiveness, suggestions and comments

### 2.2 Methodology

**Fig. 2:**
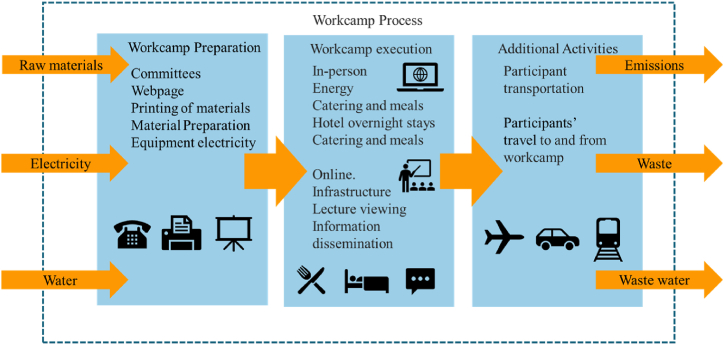
Workshop carbon emissions assessment process.

We calculated the activities, services or actions that generate CO_2_ during the conference's holding to describe the academic conference's carbon footprint [35]. In conjunction with Neugebauer et al.'s approach [24], we divided the workshop carbon footprint assessment into three parts: workshop preparation, execution, and additional activities (workshop-related travel of attendees). Phase 1, workshop preparation, includes workshop committee preparation prior to the workshop opening, including printing of workshop materials, workshop webpage creation, and preparation of materials for the workshop organisation. Phase 2, workshop execution, includes in-person energy consumption in the meeting rooms and the environmental burden associated with the participants' meals and hotel stays. The online workshop includes energy consumption during the use of infrastructure, lecture viewing, and dissemination by all participants: Phase 3, additional activities - attendee travel, including transportation to attendee workshop locations. The process included in the evaluation is shown in Fig. 2. Based on the full life-cycle workshop, the formula for the overall calculation is:

(1) $$CF=\sum_{i}^{n}MiTi$$

Where CF is the carbon footprint, M is the activity level and the amount of energy or material consumption, and T is the carbon emission factor, i.e., the amount of carbon dioxide emitted per unit of material or energy. i is the activity item, and n is the total number of items or components.

#### 2.2.1 1Workshop preparation

The workshop preparation phase includes the workshop preparation committee workshop before the workshop opening. Considering that this workshop did not collect abstracts and the prevalence of online reading, the workshop materials printing considered posters, workshop manuals, workshop schedules, and other conference printed materials as shown in the supplementary information, and the carbon emission per person in the preparation phase was calculated to be 5.07 kg.

#### 2.2.2 2workshop execution

The implementation phase of the in-person workshop was divided into three main components: firstly, the energy consumption and waste emissions from the conference room during the workshop (e.g. computer use during the workshop). The carbon emission per person in the execution phase was 22.74 kg. Second, the participants' food consumption. In this paper, we use food availability per capita in China from FAOSTAT to calculate the carbon footprint of food during the workshop and calculate the carbon emissions from meals per person per day as 4.805 kg. The final consideration is the environmental burden of the participants' accommodation, like the carbon footprint generated by the hotel or residential accommodation. Unlike ordinary conferences, workshops have a longer duration, so accommodating a carbon footprint is necessary for in-person workshops. This paper combines scholarly research on conference accommodation [26,36]. The final calculated total is 0.65 kg per person per day. The calculation is shown in the supplementary information.

The execution of the online workshop included the use of infrastructure for the participants and the energy consumption during the lecture viewing and dissemination. Combining the participants' daily work locations, working hours, and working equipment from the questionnaire and considering the long-term workshop and the hot summer season, the participants' household electricity consumption (air conditioning and another lighting, etc.) was included in the calculation. We consider the lighting time, equipment wattage and air conditioning use of attendees; with Burtscher's research on carbon emissions from online meetings [22], this paper converted the energy consumed into carbon emissions consumed with Equation (2).

(2) $$CF_{i}=\Sigma_{i}^{n}H_{W}WT_{i}$$

where CFi is the Carbon Footprint of Infrastructure, H_w_ is Working hours, W is Equipment Wattage, and T is the Carbon Emission Factor, i.e. the amount of carbon dioxide emitted per unit of material or energy. i is the activity item, and n is the total number of items or components.

The energy consumption during lecture viewing and dissemination is mainly the carbon footprint consumed by online video viewing. In this workshop, 3 scholars were invited to lecture, and 12 participants shared their recent research findings. Over 100 people participated in the online workshop to listen to and discuss the lectures, and more than 1000 views were accumulated in the live stream. The carbon footprint of their viewing and dissemination was calculated based on personal devices and network infrastructure (and servers).

The specific formula is as follows.

(3) $$CF_{C}=\Sigma_{i}^{n}D_{W}PH_{w}RCT_{i}$$

where CFc is the Carbon Footprint of Propagation, H_w_ is the number of Working days, P is the number of participants, H is the time spent online, R is the data rate, C is the electricity consumption per gigabyte, and T is the Carbon Emission Factor, i.e., the amount of carbon dioxide emitted per unit of material or energy. i is the activity item, and n is the total number of items or components.

#### 2.2.3 Additional workshop activities

The workshop's additional activities are mainly transportation activities for in-person workshop participants. The transport carbon footprint is divided into intercity and intra-city transport, where intercity transport includes air, train and intercity bus.

The formula for calculating the carbon footprint of aviation is as follows.

(4) $$E=\frac{ax^{2}+bx+c}{S\times pLF}\times \left(1-CF\right)\times cw\times \left(EF\times M+P\right)+AF\times x+A$$

where E: CO_2_-eq emissions per passenger, x: Flight Distance, S: Average number of seats (total across all cabin classes), PLF: Passenger load factor, CF: Cargo factor, CW: Cabin class weighting factor, EF: CO_2_ emission factor for jet fuel combustion (kerosene), M: Multiplier accounting for potential non-CO_2_ effects, P: CO_2_e emission factor for preproduction jet fuel, kerosene, AF: Aircraft factors, A: Airport infrastructure emissions.

where the part ax2 + bx + c is a nonlinear approximation of f(x)+LTO, LTO: Fuel consumption during landing and takeoff cycle including taxi, Short-haul is defined as x < 1500 km and long-haul as x > 2500 km. In between, a linear interpolation is used. defined as x < 1500 km and long-haul as x > 2500 km. in between, a linear interpolation is used.

The carbon footprint of trains, vehicles and buses are calculated using the energy consumed by carbon emissions by buses and cars, shown in the supplementary information.

### 2.3 Workshop carbon footprint modelling

To further consider the carbon footprint balance between in-person participation and online workshops, we constructed eight scenarios using workshop duration days and distance as independent variables and online workshops and workshop carbon footprint of different scenarios as dependent variables. This paper uses a multiple linear regression model (MLR) to construct a workshop carbon footprint model to estimate the workshop's carbon footprint under different modes. MLR is usually used to determine the causal relationship between two or more dependent and independent variables [37]. The equation is as follows.

(5) $$Y=\beta_{0}+\beta_{1}X_{1}+\beta_{2}X_{2}+\cdots +\beta_{m}X_{m}+\mathrm{e}$$

where Y is the carbon footprint of workshop β1, β2, …, βm are the various factors affecting the carbon footprint of the workshop, and β0 is a constant term. The equation indicates that the carbon footprint of the workshop can be approximated as a linear function of the various factors X_1_, X_2_, …, X_m_.

## 3 Results

**Fig. 3:**
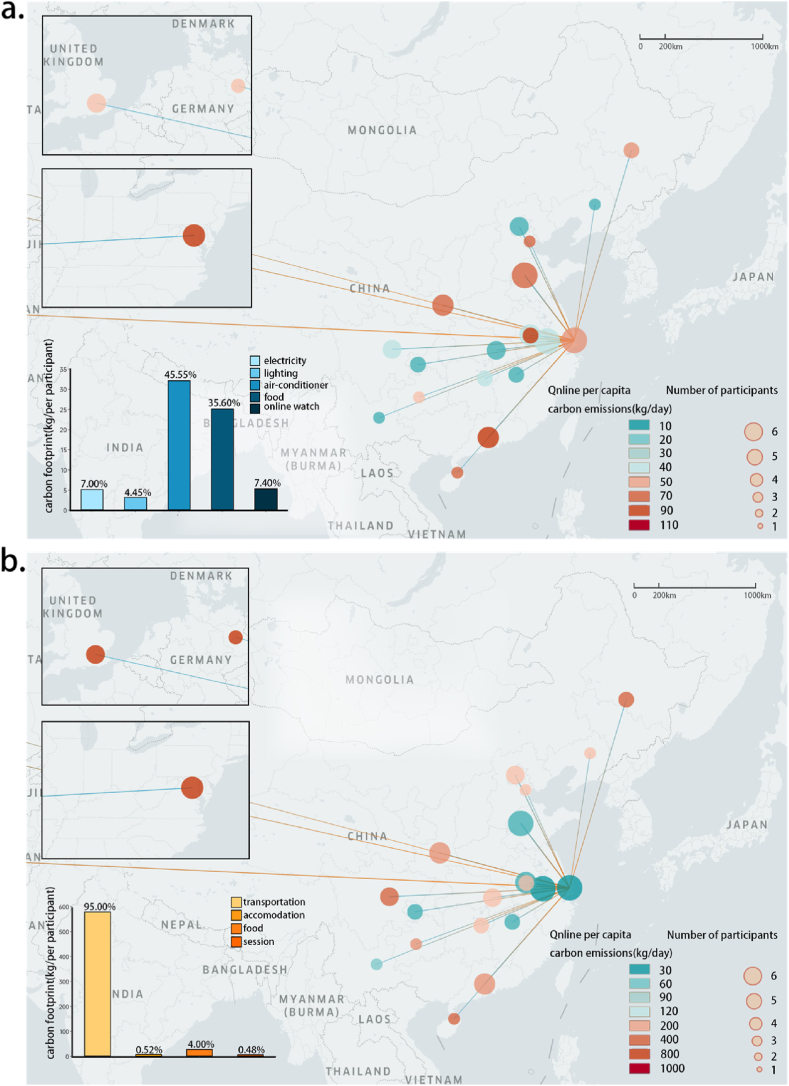
Carbon footprint of online and in-person workshops. a Geographical distribution and composition of the carbon footprint of the online workshop. b Geographical distribution and composition of the carbon footprint of the in-person workshop.

Overall, the online workshops' carbon footprint ranged from 20.63 kg to 226.98 kg (Fig. 3-a), and the in-person workshops' carbon footprint ranged from 16.47 kg to 3539.30 kg (Fig. 3-b), with a maximum difference of 7.5 times. Some multinational attendees' carbon footprint already exceeds the median global per capita carbon budget for 2030 (3.26 tonnes of CO_2_ equivalent [38]).

The transportation carbon footprint accounts for the majority (95%) of the in-person workshop carbon footprint, with food, accommodation, and services accounting for a tiny portion (less than 5%). The most significant part of the online carbon footprint is the workshop execution phase, which consists mainly of air conditioning and cooling (45%) and food consumption (35%).

**Fig. 4:**
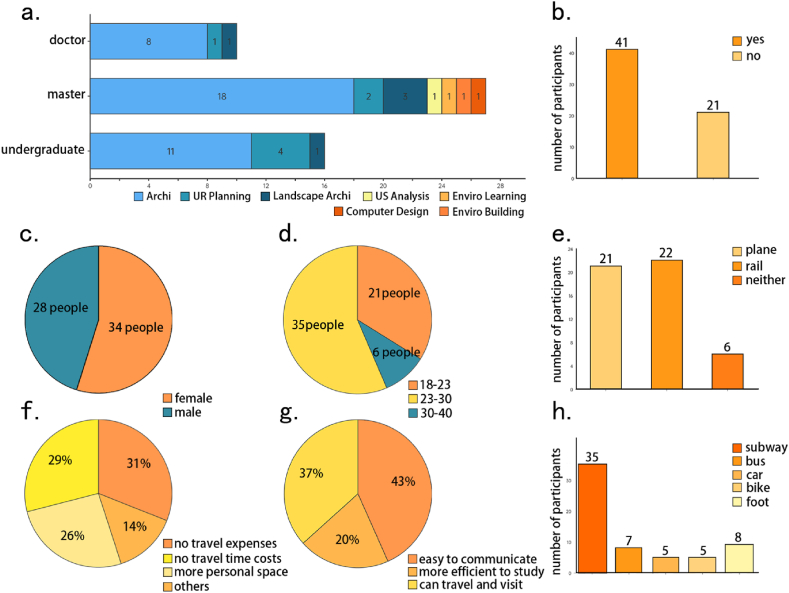
Workshop attendees. a. Distribution of attendees by education and profession. b. Attendees' willingness to choose in-person and online. c. Gender distribution of participants. d. Age distribution of participants. d. Age distribution of participants. e. Participants' choice of intercity transportation. f. Reasons for choosing in-person participation. g. Reasons for choosing online participation. h. Participants' choice of intra-city transportation.

As for the participants, nearly 70% of the workshop attendees were majoring in architecture, and most had master's degrees (Fig. 4-a). Most attendees were between 23 and 30 years old (Fig. 4-d). Regarding geographic location, participants were primarily concentrated in China (88.3%), with the Pearl River Delta and Yangtze River Delta dominating. A small number of participants were concentrated in the US and the UK. A slightly higher percentage of attendees were female (54.8%) (Fig. 4-c).

According to the survey, two-thirds (66.13%) of the attendees said they would still be willing to travel to Shanghai for the in-person workshop if there was no epidemic (Fig. 4-b). The main reasons for choosing in-person participation were the ease of communication with attendees (43.3%), the efficiency of learning in-person (36.7%), and the ability to travel and visit friends (20%) (Fig. 4-g). The remaining one-third of participants (33.87%) chose to participate online mainly because of lower travel costs (28.6%) and high travel time costs (31.4%) and because they were more accustomed to their personal space (25.7%) (Fig. 4-f). Intercity transportation options chosen by participants were mainly trains and planes, with fewer choosing cars (Fig. 4-e), and 85% of intra-city transportation options would choose the subway for travel (Fig. 4-h).

**Fig. 5:**
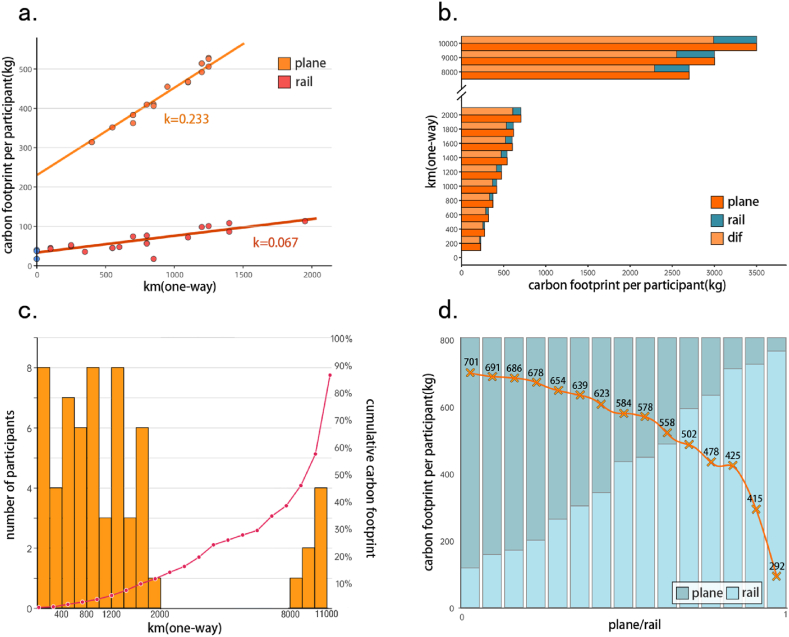
Comparison of workshop carbon footprint by transport mode. a. Comparison of carbon footprint per km by transport mode. b. Difference between online and in-person workshops for different distances. c. Distribution of distance and cumulative carbon footprint of participants. d. Comparison of carbon emissions per km by train and air. e. Comparison of carbon emissions per km by train and air. d. Comparison of carbon emissions by train and aeroplane for different ratios.

During the workshop, round-trip travel distances ranged from 200 km (Jiangsu)13 to 20,900 km (Los Angeles), with an average round-trip distance of 3714 km for participants, and Fig. 5-a shows that the carbon footprint per km (slope of the yellow dashed line) tends to be greater for travel primarily by air (slope of the blue dashed line) than by train (slope of the blue dashed line). The in-person carbon footprint shows a more pronounced regional pattern; the greater the distance, the more significant the difference. The difference is the smallest for Shanghai attendees and may be positive, while the largest for multinational attendees. The difference between provinces and cities in the online workshop was not significant. (Fig. 5-b). The majority of trips (around 90%) produced less than 1000 kg of CO_2_ emissions per trip, in contrast to cross-country flights (around 10% of all trips), which produced 55% of the total emissions of attendees. It is also in line with previous studies [21,39] (Fig. 5-c). This trend is also reflected in the ratio of planes to trains. Every 4% increase in the ratio of trains to planes will reduce carbon emissions by 6.14 kg on average (Fig. 5-d).

It has been argued that train travel has better productivity than air travel [22]. A 2-h flight usually translates into at least 4 h of travel time (including the time to the airport), leaving little time for work. On the other hand, most of the time, the train can be used productively for work, especially on modern trains equipped with power outlets and Wi-Fi connections. Of course, the main factors influencing participants' choice of transport mode are time and cost, which is also in line with our questionnaire. When choosing intercity transport, 82.93% of attendees selected air travel because it saves time and 48.78% selected train travel because of the low cost. However, with the increase in VAT and fuel surcharges on airline tickets [39], air travel prices have increased significantly, driving attendees to choose a more low-carbon mode of transport.

**Fig. 6:**
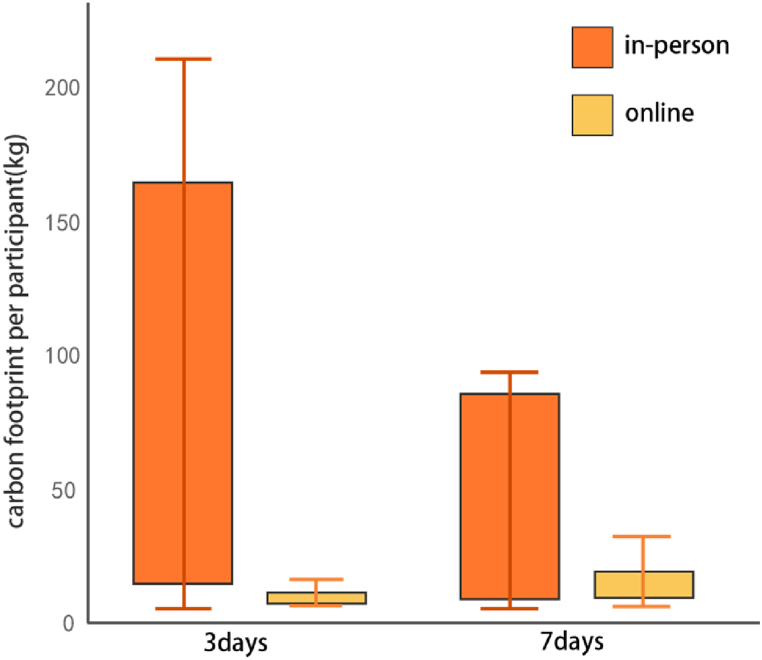
Online vs in-person carbon footprint for different numbers of days.

The number of days is also essential for online and in-person workshops(Fig. 6). Longer workshop days counterbalance the higher additional transport carbon footprint impact. 3-day workshops have a progressively lower in-person workshop carbon footprint compared to 7-day workshops. Conversely, as the number of workshop days rises, the online workshop carbon footprint gradually increases, mainly due to the cumulative increase in single-person energy consumption during the workshop execution phase.

## 4 Discussion

### 4.1 Factors affecting the workshop's carbon footprint

**Fig. 7:**
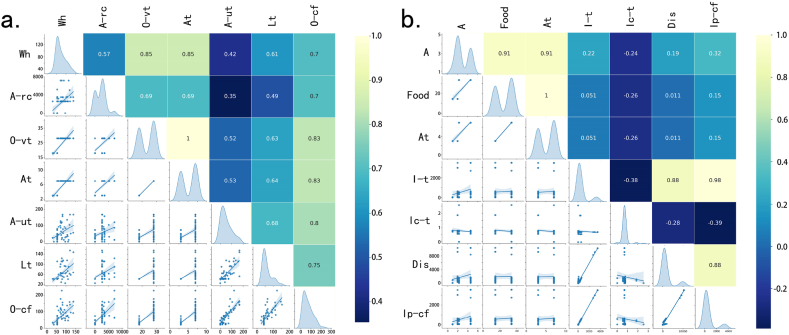
Influencing factors for online and in-person workshops. a Influencing factors for online workshops. b Influencing factors for in-person workshops. (Wh: Working hours, A-rc: Air conditioning rated cooling capacity, O-vt: Online viewing time, At: Accommodation time, A-ut: Air conditioning use time, Lt: Lighting time, O-cf: Online carbon footprint, A: Accommodation, I-t: Intercity transportation, Ic-t: Intra-city transportation, Dis: Distance, Ip-cf: In-person carbon footprint.)

We counted the impact of various elements of workshop preparation, execution and additional activities on online and in-person workshops by gender, age, education and occupation. It was found that the factors affecting online workshops were: air conditioning usage time (0.84), lighting time (0.75) and accommodation time (0.74). The most sensitive parameters for online workshops were the usage time of various types of equipment and accommodation time (Fig. 7-a). In order to reduce the carbon footprint of online workshops, we can try to use more energy-efficient air conditioning and lighting equipment or use renewable sources. The factors influencing in-person workshops are intercity transport (1.000) and distance (0.987) (Fig. 7-b), consistent with Wortzel et al. and Funke et al. [35,40]. In-person workshops are very susceptible to selecting characteristic factors, like air transport and air transport distance. Gender, age, education and occupation have little effect on workshop carbon emissions. As international workshops are events attended by researchers from all over the world, future offline workshops could consider reducing the carbon footprint by reducing distances, offering multiple workshop locations, and promoting low-carbon travel.

### 4.2 Comparison of the carbon footprint of different approaches to workshop

To further calculate the difference in carbon footprint between in-person participation and online workshop, we compare the carbon footprint of workshops under different modes. We constructed 8 in-person workshop scenarios, using train transport, all train transport and all air transport within 900 km, 1000 km, 1200 km, 1400 km, 1600 km and 2000 km, respectively, to compare the carbon footprint with that of the online workshop.

**Fig. 8:**
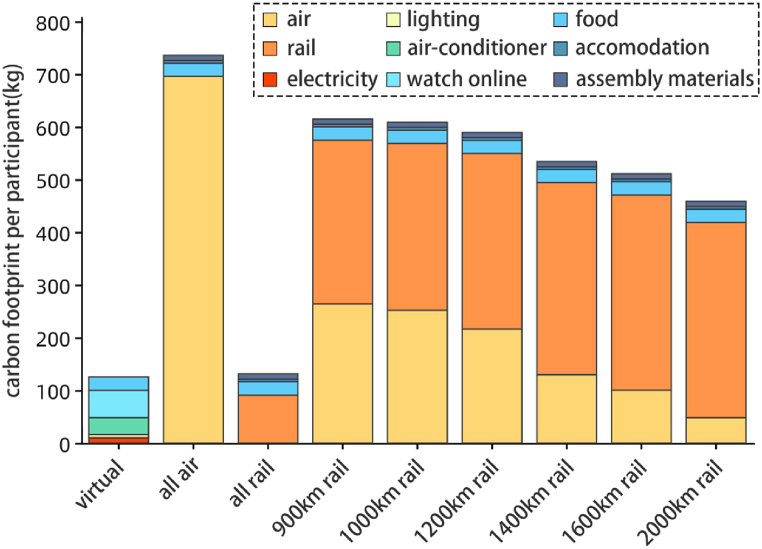
Composition of carbon footprint in different scenarios.

Fig. 8 shows the composition of carbon footprint in different scenarios, where different colours represent different processes within the LCA phase. As the number of km increases, its train's carbon footprint gradually increases, and the plane's carbon footprint decreases. On average, for every 100 km of transportation mode limit, the train carbon footprint increases by 4.5 kg and the total carbon footprint decreases by 28.41 kg.

**Fig. 9:**
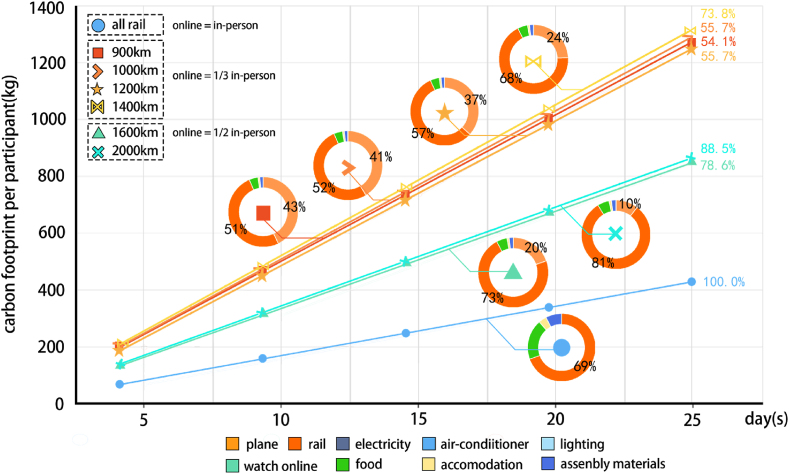
The balance of the carbon footprint of different approaches to the workshop.

As shown in Fig. 9, this paper uses a multiple regression model to construct the balance of the workshops' carbon footprint by different modes, and we can find that the distance between online and in-person workshops gradually decreases as the distance of train traffic increases. It is possible to achieve the carbon balance between online and in-person with a long time of workshop conduct and a shorter distance restriction. Specifically, with the restriction that all traffic is train traffic, 3 days of 314.87 km workshop can achieve equal carbon emissions between online and in-person, while the rest of the scenarios are not as well simulated, and there is less carbon balance between online and in-person workshops (Fig. 9). Especially when all traffic is air traffic, there are no equal carbon emissions. It is also consistent with the study by Pierce et al. [41] that we do not have a solution that can reduce emissions from air travel shortly because aviation fuels are difficult to replace with renewable energy sources. If per capita energy consumption cannot be reduced in the future, we must limit on-site workshops by cutting off flights and long-distance travel.

If the carbon gap between online and in-person workshops is restricted, the 1600 km and 2000 km scenarios can be met on days 23 and 14 when the gap is 50%, and the 900 km, 1000 km, 1200 km and 1400 km scenarios can be met on days 5, 6, 8 and 5 when the gap is 67%. The results show that even small (less than 900 km) train traffic restrictions and short-term workshops (5 days) can reduce the carbon footprint by a minimum of one-third.

Because of time, this paper suggests limiting train transportation to 900 km, 1000 km, and 1200 km and selecting different workshop transportation modes based on the different workshop days. We further hypothesise that if the workshop carry out mode is restricted. According to the distance, set 900 km, 1000 km, 1200 km, 1400 km, 1600 km, and 2000 km away using the online mode. It was found that a hybrid workshop mode could achieve a minor carbon footprint consumption for the same distance and time, and setting the online participation level at 46% could reduce the carbon footprint of the in-person workshop by 82%. If the full participation mode is pursued, limit the use of online workshops to more distant attendees. Adding 11% of online workshops can reduce carbon emissions by 39.1%. Therefore, from a carbon reduction perspective, it is beneficial to hold hybrid workshops and choose a reasonable number of days and transportation modes.

### 4.3 Feedback from participants in the online workshop

**Fig. 10:**
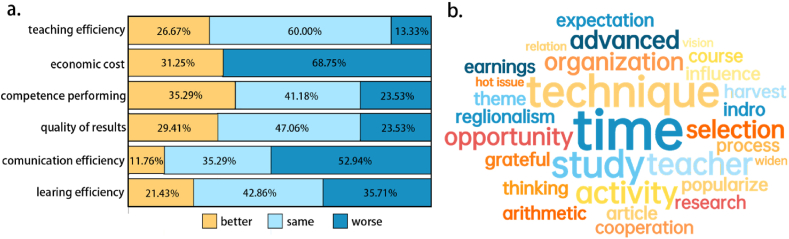
Participants' feedback on the online workshop versus the in-person workshop. a Participants' feelings about the different workshop modes. b Participants' suggestions and comments on the workshop.

Overall (Fig. 10-a), the participants received the online workshop format well, especially regarding teaching effectiveness, competency performance, quality of results, and learning efficiency, which were the same or higher than those of the offline workshops. The online workshop is very inclusive, making it easy for different participants to participate in the conference. By simply relying on an internet-based device, the online workshop can reduce the cost of conference expenses such as airfare and accommodation for poor researchers. Inclusiveness also means that researchers can participate in conferences they cannot attend due to personal issues such as family care or disability. Virtual conferences are not significantly affected by academic outcomes, which is also in line with the study by Wynes et al. [42]. As for the financial cost, most students thought offline workshops were costly. When asked about the most significant challenges they see with the online format, networking and social interaction were the parts of online workshops that participants felt needed improvement, and offline workshops can provide convenient and efficient social opportunities [35]. However, they come with a higher carbon footprint.

Meeting and travel costs are much lower for virtual and hybrid meetings compared to on-site meetings, and online workshops have a more significant advantage in terms of economic costs. It is comparable to offline work regarding teaching effectiveness, quality of competency performance and outcomes, and learning efficiency but less effective than offline workshops regarding communication effectiveness. However, Funke and Lago's study concluded that hybrid meetings could be administratively confusing because, in addition to traditional on-site meetings, organizers must pay attention to virtual meeting publishing and playback [35], which requires stable network connectivity and reliable playback devices. Offline workshops are not without advantages, as the economic income generated during the workshop and the short conference tours after the workshop will generate revenue for the city's airline, tourism and hotel industries. For example, at the annual China Urban Planning Conference, a half-day city study is added to the 3-day conference to give participants a quick overview of the urban planning situation in the region. Calculations have been made on the environmental impact of conference tourism, and it has been suggested that organizers should make sustainability a strategic priority in conference tourism, for example, by choosing low-carbon transportation. Participants need to make low-carbon choices in transportation, souvenirs and shopping, and food and beverages, among others [43].

In the feedback word cloud (Fig. 10-b), participants identified the main words as technology and learning and focused on algorithms and hot issues in the course. Participants would like to see the workshop expand its reach and introduce more relevant topics in the future. Participants preferred the online workshop because the committee could provide a video recording of the session during the workshop that could be viewed continuously after the workshop.

## 5 Conclusions

The COVID-19 pandemic has shifted much of teaching and learning from in-person to online, significantly reducing the teaching and learning process's carbon footprint and dramatically changing the current learning and education paradigm. This paper uses the LCA method to calculate the carbon footprint of online and in-person workshops and accounts for the impact of different days and modes of transport on online and in-person workshops. The results show that the transition from in-person to online workshops can reduce the carbon footprint by up to 88% and that it is possible to achieve a carbon balance between online and in-person workshops when controlling the transport mode and the workshop days. Limiting all traffic to train traffic, 3 days and more workshop time can achieve equal carbon emissions between online and in-person workshops. Adding more train traffic can reduce in-person workshops' carbon footprint and the carbon gap between online and in-person workshops. Our analysis provides a reference model for the future balance between reducing the workshop's carbon footprint and maintaining a degree of in-person interaction.

We studied the carbon trade-off between online and in-person workshops and found that limiting the mode of transport and maximum workshop time for a fixed number of days reduces the carbon emissions gap between online and in-person workshops, especially with total train transport, a 3-day workshop of 314.87 km. The carbon footprint can be reduced by at least a third, even with a small (less than 900 km) train traffic limit and a short workshop (5 days). A hybrid workshop model can achieve a minimal consumption of carbon footprint by distance, and setting the online participation level at 46% can reduce the carbon footprint of an in-person workshop by 82%. Therefore, from a carbon reduction perspective and participant perception, online workshops can reduce carbon emissions, but they are not as effective in communication and teaching. In the future, it is recommended that hybrid workshops be held, with a reasonable number of days and transport options, to achieve carbon reduction and ensure efficient learning and effective communication.

Future work could further explore ways to reduce the impact of carbon emissions and environmental workshops impact, for example, by promoting effective practices in online workshops such as energy savings from heating and other residential electricity use (like air conditioning, lighting, electronics and appliances), reducing food waste, and improving energy efficiency in the ICT sector. Environmental (air conditioning, lighting) electricity consumption is also a sensitive parameter, accounting for about half (45%) of the online workshop's carbon footprint. Choosing a more low-carbon transportation option for in-person workshops and extending the workshop duration can further reduce the carbon footprint of both online and in-person workshops, providing a sustainable carbon reduction policy for future workshops.

## Declarations

### Author contribution statement

Na An: Conceived and designed the questionnaire; Collected and processed the data, and Wrote the paper. Chenyu Huang: Collected and processed the questionnaire data; Analysed the data, and Wrote the paper and Corrected proof. Yanting Shen: Analysed the data; Wrote the paper. Jinyu Wang: Analysed the data; Wrote the paper. Jiawei Yao: Conceptualised and designed the questionnaire; Analysed and interpreted the data; Overall Supervision of the Study Performed. Philip F. Yuan: Writing - Review & Editing, Supervision.

### Funding statement

This work was supported by the 10.13039/501100001809National Natural Science Foundation of China (52278041); the Shanghai Municipal Science and Technology Major Project (2021SHZDZX0100) and 10.13039/501100012226Fundamental Research Funds for the Central Universities.

### Data availability statement

Data will be made available on request.

## Declaration of competing interest

The authors declare that they have no known competing financial interests or personal relationships that could have appeared to influence the work reported in this paper.
