# Supplementary material for: Challenges of carbon emission reduction by the workshop education pattern
Source: Heliyon. 2023 Feb 10;9(3):e13404. doi: 10.1016/j.heliyon.2023.e13404 (PMC9911162; doi:10.1016/j.heliyon.2023.e13404)
Supplement: Multimedia component 1 [file mmc1.pdf]

同济大学建筑与城市规划学院
TONGJI UNIVERSITY CAUP
COLLEGE OF ARCHITECTURE AND URBAN PLANNING TONGJI UNIVERSITY

College of Architecture and Urban Planning, Tongji University
1239 Siping Road, Yangpu District, Shanghai 200092, P.R. China
Tel: 021-65982345

To Whom It May Concern,

I am AnNa, a PhD student in the class of 2020(No.2010255) at Tongji University. I am participating in the 2022 DigitalFUTURES International Workshop for Architecture Education project. Our data source is a questionnaire collection that includes respondents' basic information, transport information and environmental information, such as the geographical location, source institutions, primary environmental surveys, daily energy consumption surveys and suggestions for improvement. All participants have given their consent to participate in this study. All data do not involve sensitive information such as respondents' names; only Workshop's daily consumption is counted for further research purposes, hereby applied for in hopes of approval.

Sincerely,

Applicant's signature An Na

Advisor's signature Jiawei Yao

Date 2022.6.20

College of Architecture and Urban Planning,
Tongji University
